# Supplementary figures and images for: S. cerevisiae Srs2 helicase ensures normal recombination intermediate metabolism during meiosis and prevents accumulation of Rad51 aggregates
Source: Chromosoma. 2019 May 9;128(3):249–65. doi: 10.1007/s00412-019-00705-9 (PMC6823294; doi:10.1007/s00412-019-00705-9)

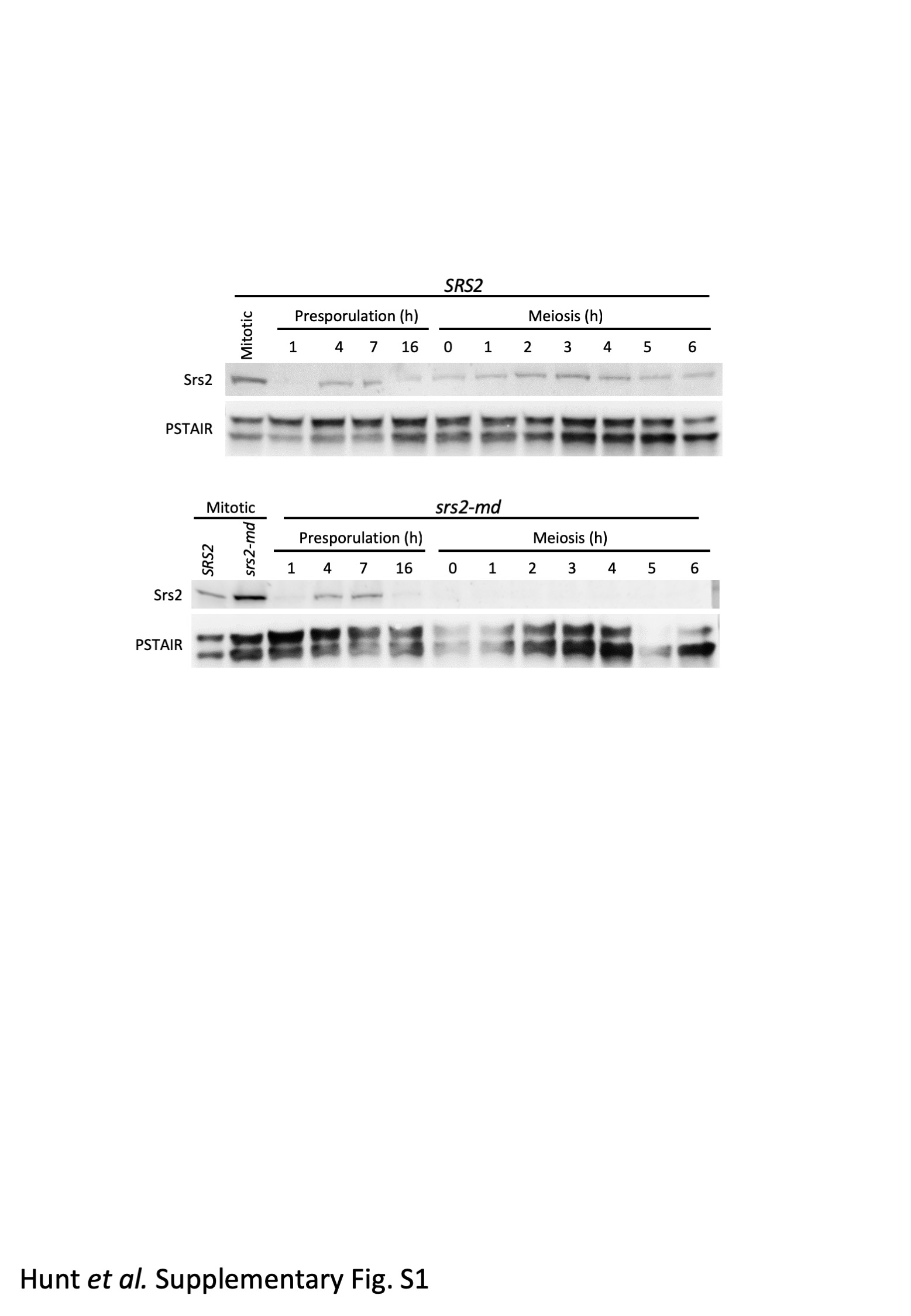

Supplement: Supplementary file 1 — Srs2 loss during meiosis in srs2-md strains. Protein samples from mitotic (YPAD overnight), premeiotic (BYTA) or sporulation (SPM) cultures of SRS2 or srs2-md strains were displayed on SDS-PAGE gels, transferred to nitrocellulose and probed with anti-Srs2 or anti-PSTAIR as described in ‘Materials and methods’. Cells were shifted to SPM after 17 h of presporulation growth in BYTA. (JPEG 163 kb) [file 412_2019_705_MOESM1_ESM.jpeg]

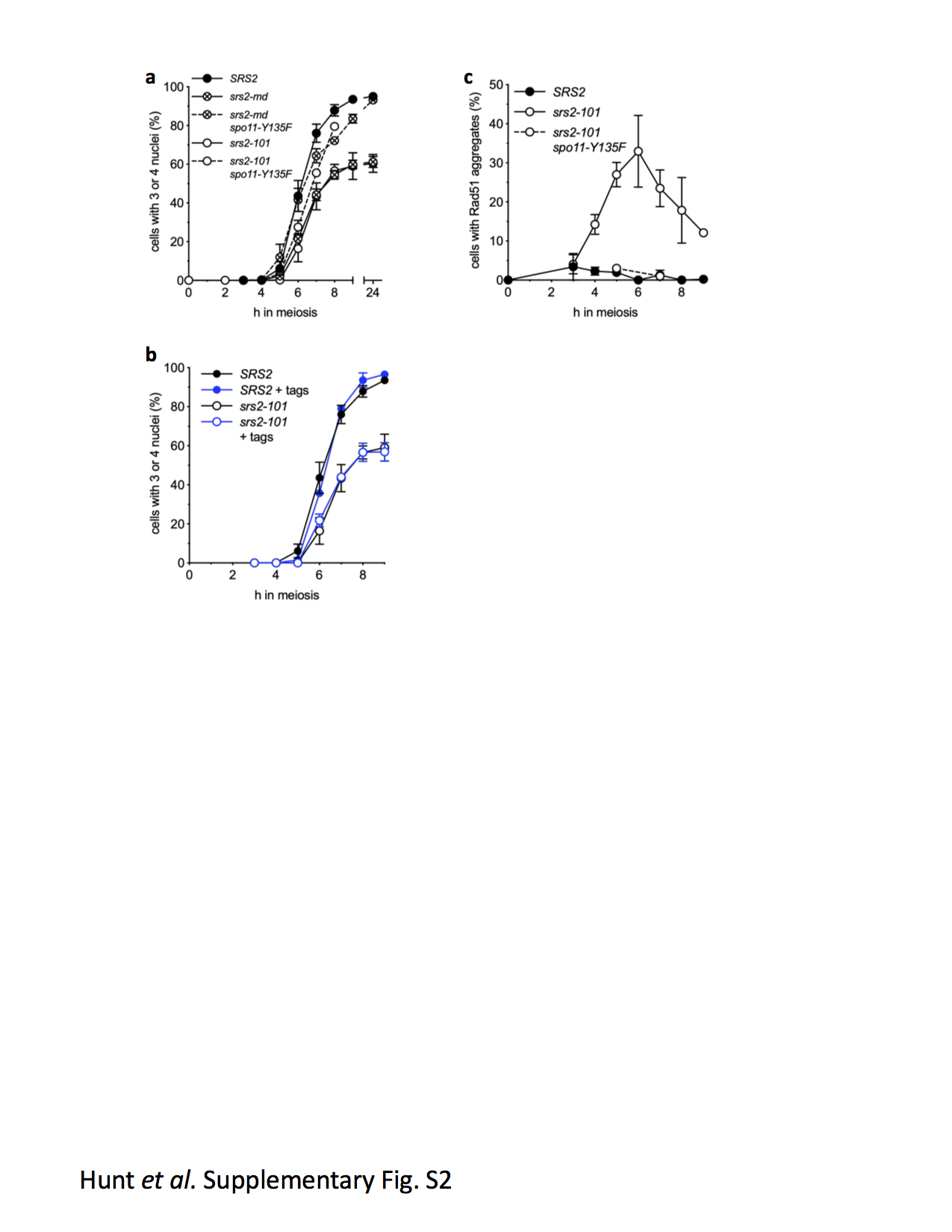

Supplement: Supplementary file 2 — a Progression defects in srs2 mutant cells require Spo11-induced double strand DNA breaks. While srs2-101 and srs2-md mutants show a failure to complete meiosis II, srs2-101 spo11-Y135F and srs2-md spo11-Y135F double mutants, which fail to form meiotic DSBs, restore progression to near wild-type levels (srs2-md spo11-Y135F, n = 3; srs2-101 spo11-Y135F, n = 2; data for SPO11 strains from Fig. 1). b Progression through meiotic nuclear divisions, expressed as percent of cells completing meiosis II, is similar in the presence (+ tags) or absence of CNM67-mCherry and GFP-TUB1 (SRS2 + tags, n = 2; srs2-101 + tags, n = 2; error bars—range; data for untagged strains from Fig. 1). c Rad51 aggregates form in srs2-101 mutants. (srs2-101, n = 3; srs2-101 spo11, n = 3; data for SRS2 from Fig. 4; error bars—standard error of the mean) (JPG 222 kb) [file 412_2019_705_MOESM2_ESM.jpg]

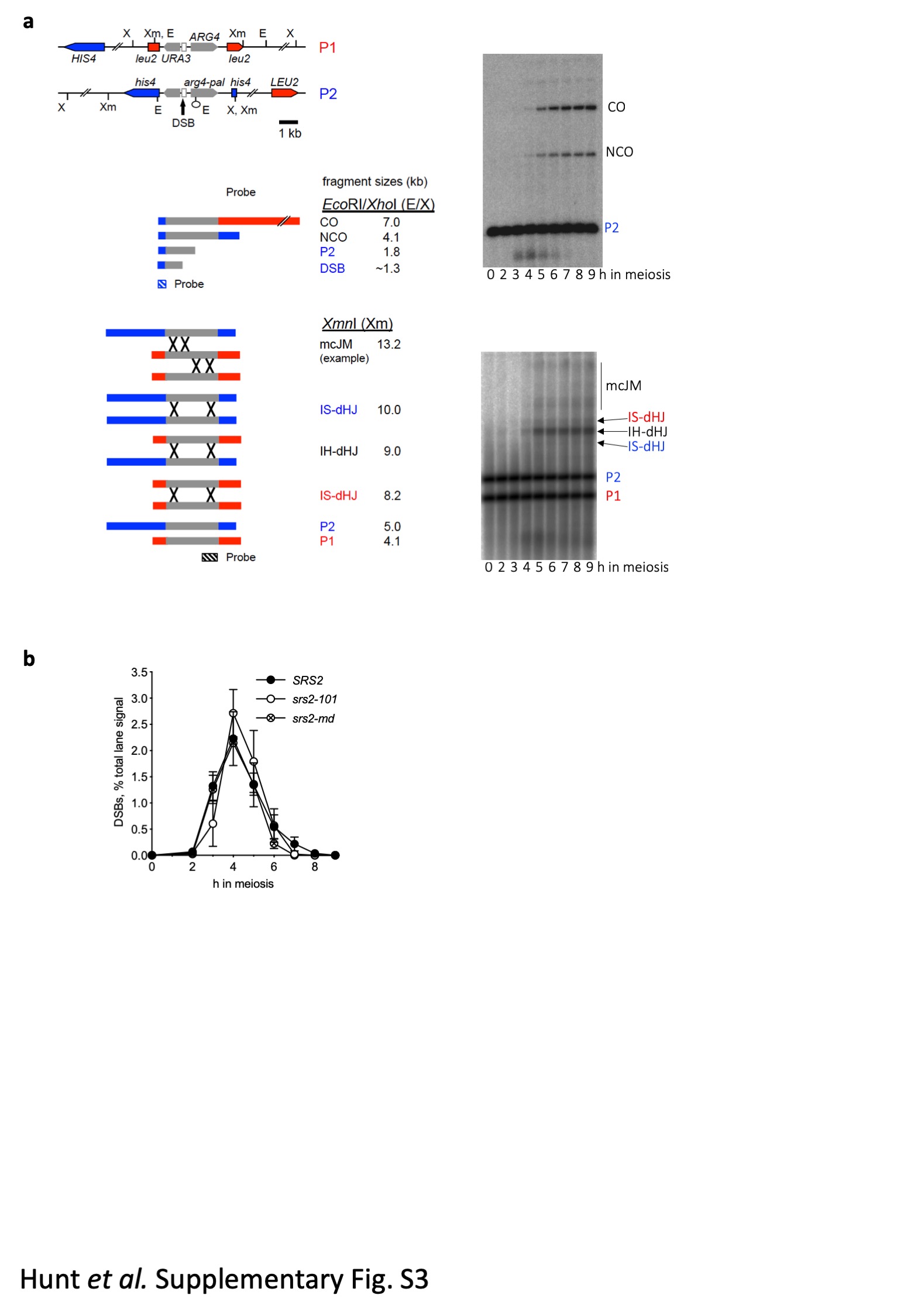

Supplement: Supplementary file 3 — a Recombination reporter system used to detect recombination intermediates in XmnI digests and products in EcoRI/XhoI double digests (Jessop et al. 2005; Jessop and Lichten 2008). Grey arrows—URA3 and ARG4 genes; white box—63 nt telomere repeat sequence; vertical arrow—meiotic DSB hotspot. Inserts are at LEU2 (red) on one chromosome III and at HIS4 (blue) on the other. arg4-pal is an EcoRI-marked palindrome insertion for scoring noncrossovers. Relevant restriction sites are indicated. EcoRI/XhoI digests detect DSBs, NCO and CO products. XmnI digests detect interhomologue (IH-dHJs) and intersister double Holliday junctions (IS-JMs) and multichromatid JMs (mcJMs, only one example of many possible is shown). Schematic reproduced from Kaur et al. (2015); illustrative Southern blots are from an SRS2 diploid. b)DSB dynamics are not altered in srs2 mutants. DSBs were measured on Southern blots of EcoRI/XhoI digests of DNA from SRS2 (n = 5, data from De Muyt et al. 2012; Kaur et al. 2015 and one additional replicate; error bars denote SEM), srs2-101 (n = 5; error bars denote SEM) and srs2-md (n = 2; error bars denote range). (JPEG 211 kb) [file 412_2019_705_MOESM3_ESM.jpeg]
